# Corneal allograft endothelial cell replacement represents a reparative response to transplant injury

**Published:** 2009-04-03

**Authors:** Nianqiao Gong, Uwe Pleyer, Thomas Ritter, Erich Knop, Xiaoping Chen

**Affiliations:** 1 Institute of Organ Transplantation, Tongji Hospital, Tongji Medical College, Huazhong University of Science and Technology, Wuhan, China; 2 Department of Ophthalmology, Charité–University Medicine Berlin, Germany; 3 Regenerative Medicine Institute, National University of Ireland, Galway, Ireland

## Abstract

**Purpose:**

To elucidate the injury of corneal allograft endothelial cells (ECs) upon rejection and the subsequent replacement process of the cells.

**Methods:**

The corneal transplantation model in an major histocompatibility complex (MHC) class I/II disparate Dark Agouti (DA)-Lewis combination was used. The rejection kinetics was observed in 16 cases in which the corneal opacity grade was recorded after grafting and after the onset of rejection. Four normal corneas and four allografts were subjected to EC staining to investigate the EC integrity in cases of rejection. Furthermore, a series of rejected allografts were examined and the EC integrity compared at one week, three weeks, three months, and six months after the onset of rejection.

**Results:**

All corneal allografts were rejected, resulting in EC integrity loss. However, the allografts recovered transparency around 18 days after the onset of rejection with repaired endothelium by regenerative ECs. Moreover, although the whole endothelium would be fully recovered after rejection, the ratio of regenerative EC density reached only half of normal levels as long as six months after the transplant.

**Conclusions:**

Corneal allograft EC replacement represents a reparative response to transplant-related injury.

## Introduction

With more than 50,000 procedures per year in the US, the cornea is the most commonly transplanted solid tissue [1-3]. An endothelial cell (EC) monolayer covers the inner side of the endothelium, and its most important function is to pump the infiltrating liquid out of the stroma and into the anterior chamber to maintain corneal transparency. In cases of corneal transplantation, immunological rejection remains one of the main obstacles for a well functioning allograft, and ECs are the critical target of rejection. The EC loss results in stroma edema and a decrease of cornea graft transparency, which is an indication of rejection onset.

For corneal allografts, although the EC monolayer is one critical compartment, few studies have focused on its pathological response to rejection. One explanation is that the opacity of rejected human corneas cannot be reversed [4], a situation which prevents researchers from carrying out a sufficient investigation of the underlying pathophysiology. But in some animal models, a return of corneal clarity has been noted following rejection [5].

Although the interesting phenomenon of corneal allograft transparency recovery has been observed, the EC regenerative progress, including the absolute numbers of the cells and their ratio in comparison with normal corneas, has not yet been fully investigated. In the present study, we used a rat corneal transplantation model to detail the kinetics of corneal allograft EC replacement in the hopes of furthering our exploration into corneal protective therapy.

## Methods

### Animals

Inbred female rats of Dark Agouti (DA, RT.1A^av1^) and Lewis (RT.1A^1^) strains weighing 200–250 g were obtained from Charles-River (Kisslegg, Germany). Lewis rats served as recipients of DA grafts, which are major histocompatibility complex (MHC) class I/II disparate. All of the animals were housed in wire-bottomed cages with controlled light/dark cycles, fed with a standard laboratory diet, and given free access to tap water. Animals were handled in accordance with the National Institute of Health “Guide for the Care and Use of Laboratory Animals” and the German guidelines on the use of animals in research (Title: Berliner Senatsverwaltung).

### Corneal transplantation and definition of graft rejection

Orthotopic corneal transplantations were performed as reported previously [6,7]. Briefly, all animals were anaesthetized by an intramuscular injection of a mixture of ketamine (90 mg/kg, Ketavet; Pharmacia GmbH, Erlangen, Germany) and xylazine (7.5 mg/kg, Rompun 2%; Bayer Vital GmbH, Leverkusen, Germany) diluted in saline during the surgical procedure. Prior to surgery, 1% atropine sulfate drops (Ciba Vision, Wefling, Germany) were topically applied to dilate the pupil. The recipient and donor right cornea were trephined with a 3.0 mm or 3.5 mm trephine, respectively, and excised using Vannas scissors. The donor graft was sutured into the recipient bed using a running suture (10–0 Mersilene; Ethicon, Hannover, Germany). The suture was not removed. After transplantation, antibiotic ointment (Ofloxacin, Floxal™; Mann Pharma, Berlin, Germany) was applied immediately to the eye. Animals with surgical complications such as intraocular hemorrhage or cataract were excluded. Corneal opacity as an indicator of corneal endothelial function and of graft endothelial injury was evaluated daily. Corneal opacity was graded as follows: 0, completely transparent cornea; 1, slight corneal opacity but iris vessels easily visible; 2, moderate corneal opacity, iris vessels still visible; 3, moderate corneal opacity, only pupil margin visible; 4, complete corneal opacity, pupil not visible. Grades of 3 or higher were diagnosed as rejection onset [6,7].

### Experimental groups

First, 16 DA-Lewis transplants were in one group for observation of rejection and recovery kinetics, in which the corneal opacity grade was recorded daily after grafting and rejection onset. We also employed an isograft control group in which six Lewis-Lewis corneal transplants were performed and the fate of the isografts was observed for four weeks to identify if the allograft transparency decrease was attributable to surgical trauma or alloimmune response. To investigate the EC integrity under rejection, four normal DA corneas, which were used as controls, were collected for EC staining, and four additional grafts, which were diagnosed as rejection onset, were also collected and stained. To clarify EC replacement after rejection, another series of animals with rejected grafts were housed. The animals were then divided into four groups according to the different time points (one week, three weeks, three months, six months) after rejection onset when they were sacrificed for the graft EC integrity by staining with four animals included in each group.

### Recovery of corneal allograft opacity grade after rejection

After rejection onset, the opacity grade of the corneal allografts was recorded daily until all of them reached grade 0 in the 16 DA-Lewis transplants. The recovery duration (in days) from rejection onset (grade 3 or 4) to grade 2 was counted in each animal. The duration from rejection onset to grade 0 (which means a full-functioning clear graft) was also documented. The opacity grade was recorded again three and six months after rejection onset.

### Staining of corneal endothelial cell

Both corneal allografts and normal corneas were harvested. Analysis of DNA fragmentation (Terminal deoxynucleotidyl Transferase Biotin-dUTP Nick End Labeling [TUNEL] assay; Boehringer, Mannheim, Germany) on the cornea button was used for the staining of corneal endothelial cells. In brief, the corneas were fixed by acetone at 4 °C for 10 min. Fifty microliters of TUNEL reaction mixture (2 μl enzyme solution and 48 μl label solution) was added to each sample and incubated in a humidified chamber for 60 min at 37 °C. A 50 μl converter peroxidase (POD) was then added to each sample under the same conditions for 30 min. To develop the color, a 50 μl mixture of diaminobenzidine (DAB)-substrate solution was added to each sample and incubated for 5 min at room temperature. Normal corneas were stained without any enzyme solution and were used as the negative controls. The staining was analyzed with a light microscope. ECs were easily identified with the nuclear staining. Slight brown nuclear staining was seen as normal background and black nuclear staining as positive apoptosis staining.

### Endothelial cell distribution in normal corneas and rejected allografts

The EC integrity in the normal corneas and rejected transplants provides a baseline for further analysis of EC alteration. Four normal DA corneas and four allografts diagnosed as rejection onset were collected and stained for counting cell number and observing cell shape and distribution.

### Regenerative endothelial cells recovering the graft endothelium following rejection onset

Four corneal allografts at each different time point (one week, three weeks, three months, and six months after rejection onset) were collected for EC staining. The recovered area was evaluated by percentage to describe the reparative process. The EC density was calculated by numbers/mm^2^, and cell ratio (allograft to normal DA cornea) was expressed as a percentage.

### Statistical analysis

Graft survival was presented as median±standard error (MST) using the Kaplan–Meier survival method. The other data were calculated using Statistical Package for the Social Science (SPSS) 11.0 (SPSS Int., Chicago, Illinois) by one-way ANOVA and χ^2^ test. A statistically significant difference was defined as p<0.05 .

## Results

### All Lewis recipients rejected their Dark Agouti corneal allografts featured with endothelial cell loss

**Figure 1:**
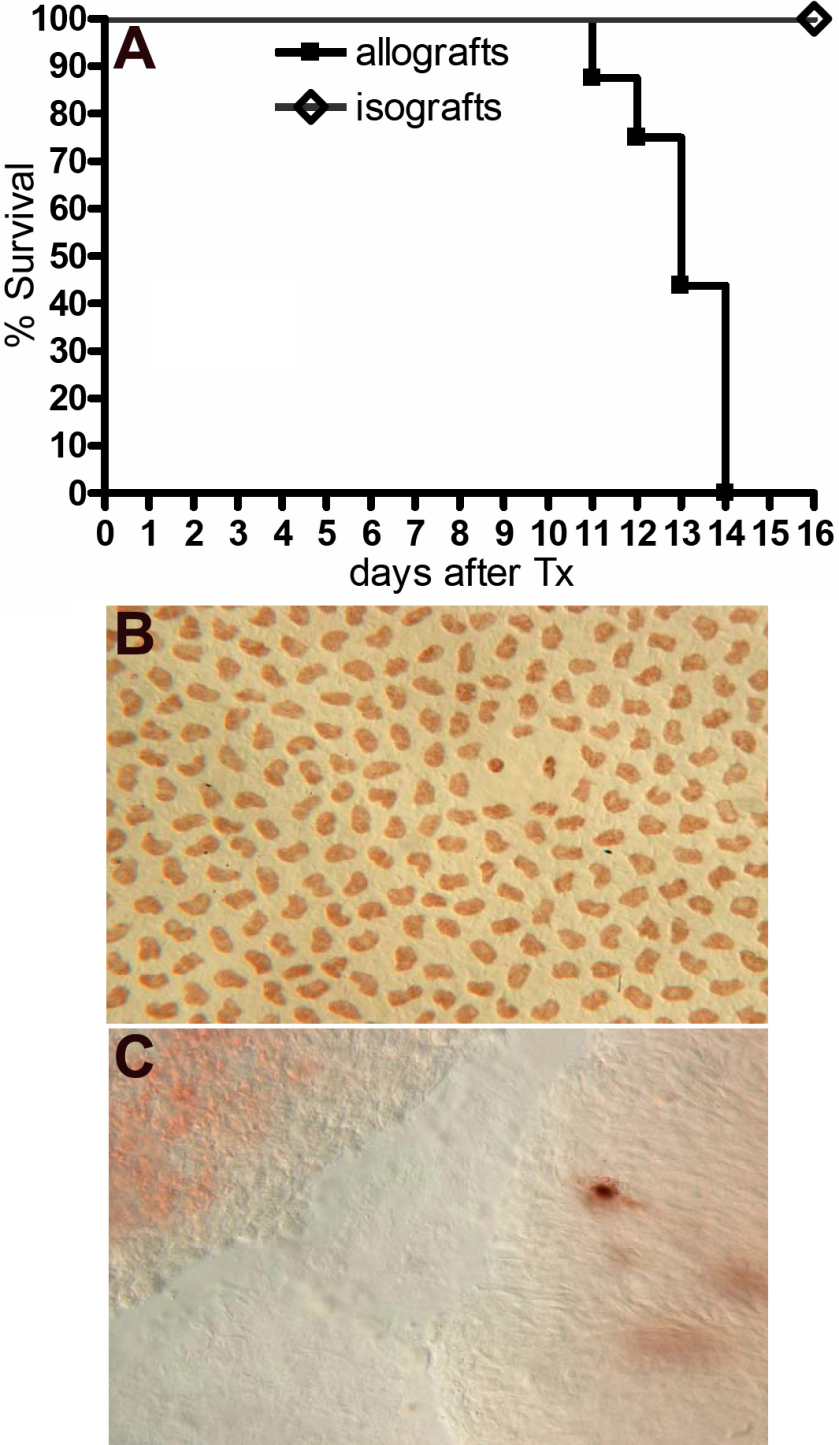
The effect of rejection upon corneal allografts. **A**: The rejection kinetics of corneal transplantation in the DA-Lewis combination is shown. Sixteen Lewis rats served as recipients of DA allografts. The opacity grade 3 or 4 is defined as rejection onset. The rejection happened in all allografts from day 11 to day 14 with an MST of 13.1±0.3 days (n=16). Meanwhile, no rejection was observed in any of the isografts (n=6). **B**: The EC staining in a normal DA cornea is displayed. The cells were distributed regularly, and their nuclei exhibited similar shape (magnification 400X). **C**: The EC staining in rejected corneal allograft is shown. All ECs were lost except the remaining apoptotic cell (400X).

To investigate the rejection kinetics of the MHC class I/II disparate DA-Lewis corneal transplant, 16 Lewis rats were employed as the recipients of DA corneal grafts. No treatment was given to either the donors or the recipients. During the first week after transplantation, the grafts had slight stroma edema but then recovered clarity within one week. Afterward, their opacity grade increased gradually to 3 or 4, which was defined as rejection onset. The rejection kinetics of these allografts is shown in Figure 1A. The rejection happened in all allografts from day 11 to day 14 (MST 13.1±0.3 days, n=16). In the isograft control group, except when the slight edema happened due to surgical trauma, all transplants retained clarity in the observation of four weeks, showing that the opacity grade increase was attributable to the immunological response.

To clarify the integrity of the allograft ECs under rejection, four normal DA corneas and four additional allografts on the day of rejection were collected for EC staining. The staining photos are shown in Figure 1B,C, respectively. The ECs were distributed regularly on the normal cornea endothelium. In contrast, all ECs on the rejected corneas were shed except for few remaining apoptotic cells.

### Corneal allograft transparency gradually recovered after rejection onset

**Figure 2:**
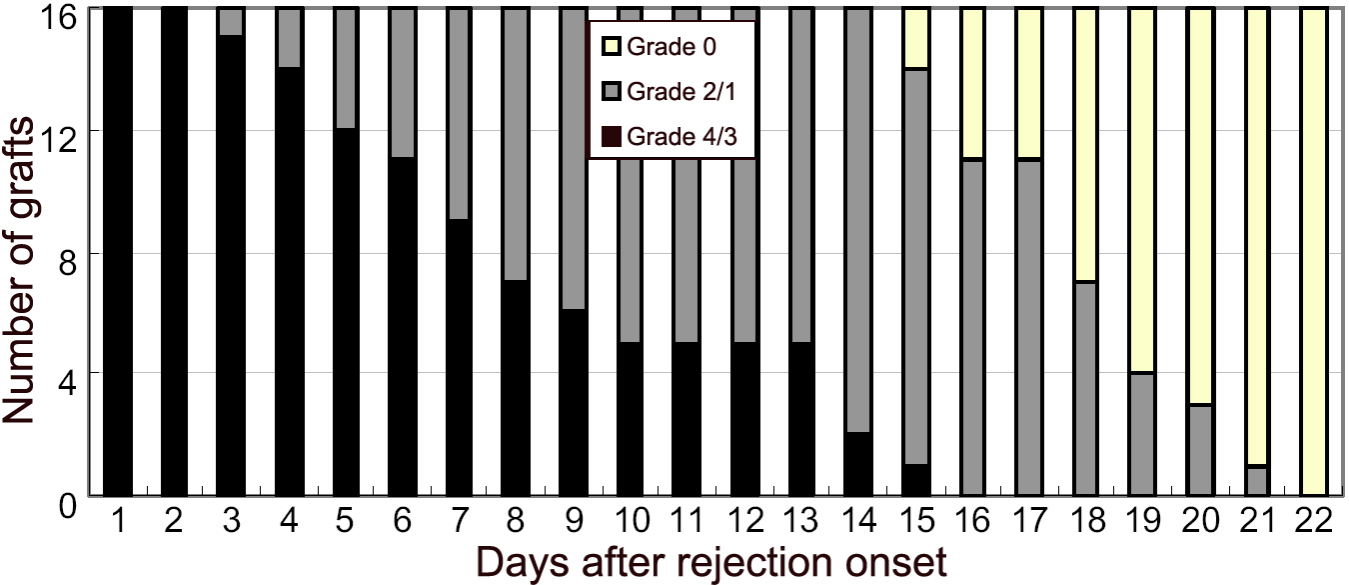
Recovery process of the corneal allograft transparency after rejection onset. Following rejection onset, the number of allografts was documented until all of them regained completely clarity up to 22 days based on their opacity grade. Grade 4/3 (black) represents grafts rejected, grade 2/1 (gray) represents grafts recovering, and grade 0 (blank) represents grafts recovered.

We continued our observation of the graft opacity after rejection among the 16 animals. It was a constant phenomenon that all of the grafts would recover clarity gradually from grade 4/3 back to grade 0, which indicated a well functioning EC layer covering the endothelium. Among all 16 animals, the longest time for clarity recovery took 22 days. The alteration of opacity grade of these allografts was shown in Figure 2. The opacity grade of the transplants was recorded daily for 22 days after rejection, and the numbers of transplants with opacity grade 4/3 (rejection), 2/1 (recovering), and 0 (recovered) used to document the recovery process were also recorded. From rejection onset to grade 2 (a key step indicating the beginning of function recovery), the duration was 8.9±4.1 days. From rejection onset to grade 0 (full-functioning), the duration was 18.3±2.2 days. Once the opacity grade had recovered, the cornea maintained stable clarity during the whole observation up to six months.

### Corneal allografts with recovered transparency were covered by newly generated endothelial cells

**Figure 3:**
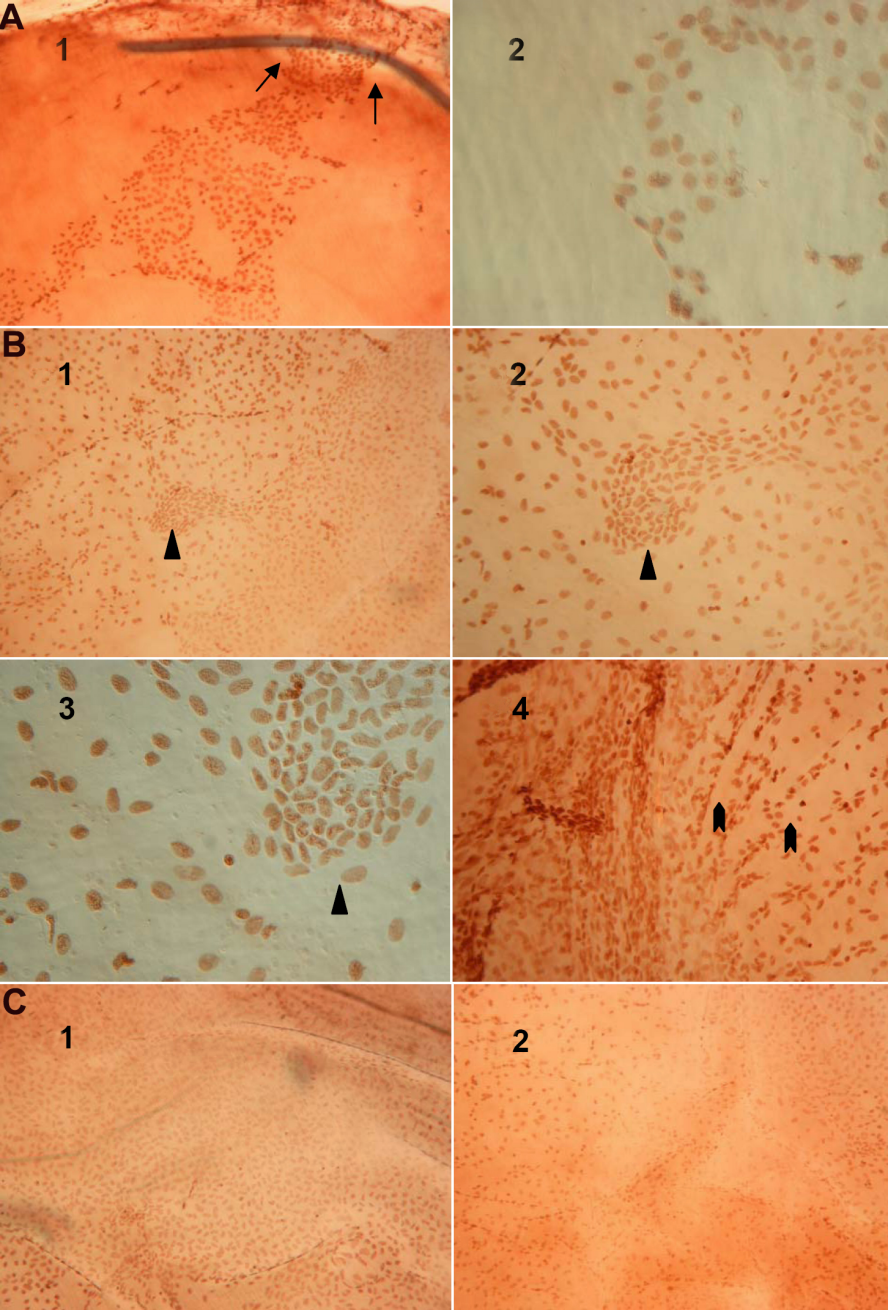
The process of corneal allograft EC replacement following rejection. **A**: The DA corneal allograft EC staining one week after rejection onset is displayed. ECs were covering part of the graft endothelium (**A**1; magnification 100X). The arrows show that the immigrating cells were crawling across the suture.ECs were covering part of the graft endothelium (**A**2; 400X). **B**: The DA corneal allograft EC staining three weeks after rejection onset is shown. The whole endothelium was covered by ECs where the distribution and cell nucleus shape were not as regular and the cell number was less than the normal level (**B**1-**B**3). The triangle shows a germinal center-like structure (**B**1,100X; **B**2: 200X; **B**3: 400X). The bold arrow shows the cells were growing radially with an active proliferation state (**B**4; 100X). **C**: The DA corneal allograft EC staining three and six months after rejection onset is displayed. Three months after rejection onset, a relatively intact ECs layer is shown, although not yet perfect (**C**1; 100X). Six months after rejection onset, a relatively intact EC layer was shown (**C**2; 100X).

Our above mentioned data prove that rejection results in losing all of the graft ECs. To further identify the EC status on graft endothelium after rejection, a series of rejected grafts from different time points (one week, three weeks, three months, and six months after rejection onset) were collected for staining. One week after rejection onset, ECs were detected covering part of the graft endothelium (Figure 3, A1,A2) while the corneal opacity grade was decreasing. Three weeks after rejection, the whole endothelium was covered by ECs, although the distribution and cell nuclear shape were not as regular as they normally would be and the amount was also less than normal (Figure 3, B1,B2,B3,B4). The opacity grade reached 0 or 1, indicating full EC layer function for the cornea. Three months and six months after surgery, the corneas retained clarity and had a relatively intact EC layer (Figure 3, C1,C2), although not yet to the same extent as the normal corneas (Figure 1B).

### The regenerative endothelial cells repaired the transplant injury but presented a density less than normal

**Figure 4:**
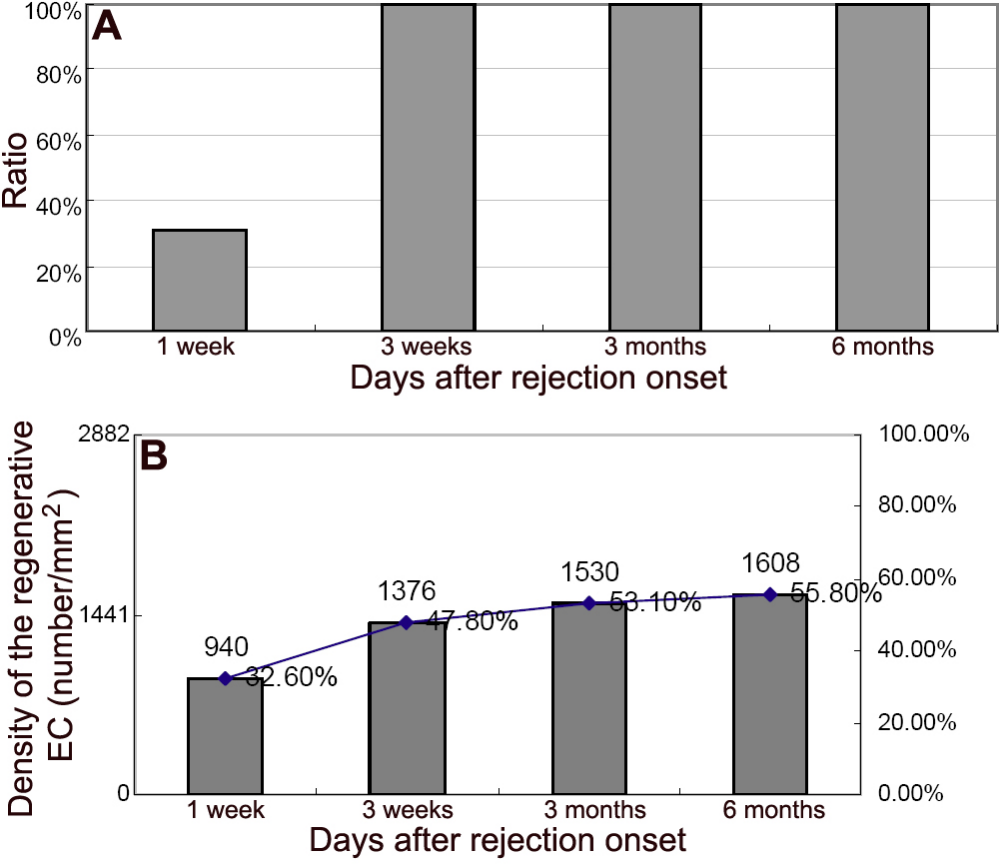
The kinetics of regenerative ECs covering the endothelium. **A**: The percentage of endothelium covered area at different time points after rejection onset is shown in the chart. The area of recovered endothelium was calculated after one week, three weeks, three months, and six months. From three weeks after rejection onset, the recovered area reached 100% of the normal level. **B**: The density and percentage of the regenerative ECs compared with normal cornea is shown in the second chart. The EC density of a normal DA cornea is 2882/mm^2^. At the different time points, the density and percentage of the regenerative ECs were calculated. The density of the regenerative ECs was 32.6% of the normal level at one week after rejection onset, and then gradually improved. However, inconsistent with the area recovery, the density reached only 55.8% of the normal level up to 6 months after rejection onset.

We then analyzed the regenerative EC’s distribution at different time points after rejection onset. By staining the series of rejected grafts at different time points (one week, three weeks, three months, and six months), the percentage of the endothelium covered area was calculated. They were 31%, 100%, 100%, and 100% at one week, three weeks, three months, and six months, respectively (Figure 3, A1,B1,C1,C2, and Figure 4A). Furthermore, EC density of the normal DA corneas and corneal allografts were calculated and compared. In the normal corneas, the EC density was 2882/mm^2^ while it was 940/mm^2^, 1376/mm^2^, 1530/mm^2^, and 1608/mm^2^ in the allografts at one week, three weeks, three months, and six months after rejection onset, respectively. The EC densities were 32.6%, 47.8%, 53.1%, and 55.8% of the normal level at one week, three weeks, three months and six months, respectively, as shown in Figure 4B.

## Discussion

According to their species, the corneal allografts exhibit different outcomes in response to rejection. Human corneal EC loss leads to graft failure in clinic while in mice, 50%–60% of recipients do not undergo any rejection. To find clues for promoting the efficacy of human allografts, a stable corneal rejection model is needed to investigate the underlying mechanism of rejection and graft opacity. In the present study, we introduced the DA-Lewis combination in which all grafts were rejected within two weeks of transplantation as shown in Figure 1A. The rejection was diagnosed by a marked increase of opacity grade, an indicator that has been adopted widely in this study area [8-10]. To further verify if the allograft opacity was indeed due to rejection, we used one isograft group as the control, which excluded the influence of immunological factors, and the result showed that the surgical trauma only induced a temporary slight edema after a successful surgery.

In this study, rejection destroyed the whole EC layer (Figure 1B) so that donor ECs did not exist afterward and the corneal transplants became opaque. But interestingly, the graft transparency recovered gradually. First, the grafts began to regain clarity around one week after rejection, a fact established by the observation that the opacity grade decreased to 2. Then, the grafts reached total transparency around 18 days after rejection. The time to transparency recovery represents a process of endothelium repair. An extended observation of up to six months showed that this recovery exhibited a stable status, indicating that the corneal structure was reconstructed with full function.

We then checked the EC status in the process of transparency return and found that the dynamics of the EC recovery were time-dependent. One week after rejection, the regenerative ECs covered one-third of the area of the endothelium with a similar value of EC density ratio. Meanwhile, the graft function obtained a substantial improvement from opaque to opacity grade 2. From the time point of three weeks after rejection, the graft recovered transparency, and the endothelium was fully recovered in the whole observation up to six months. However, the EC density showed an increase inconsistent with the area recovery, exhibiting only half the level of the normal even up to six months after rejection onset. Meanwhile, these ECs exhibited irregular features in both their cell morphology and distribution. Subtler factors influencing the EC regeneration and proliferation and the reparative response against the transplant-related injury must surely exist, a consideration that should be further explored. 

It is likely that the regenerative ECs developed mainly from the host endothelium. First, the allograft ECs were lost due to rejection, therefore no donor-derivation existed as shown in Figure 1C. Furthermore, the staining results showed three interesting histological observations. First, the proliferating cells were crawling across the suture as shown in Figure 3, A1. Second, the proliferating cells located together to generate a germinal center-like structure with irregular nuclear size (Figure 3, B1,B2,B3). Third, the proliferating cells were growing radially, indicating an active proliferation state as shown in Figure 3, B4. Although without direct MHC haplotype evidence, it is suggested that the regenerative cells are developed from the host’s adjacent corneal endothelium. In other solid organ allografts, it has been hypothesized that EC precursors are recruited from a variety of sources depending on the severity and duration of injury. During limited damage, neighboring ECs will provide sufficient repair potential. More severe damage may signal in-growth of ECs from adjacent host tissue, and severe damage with a full rupture of layers will lead to recruitment via circulation [11-13]. In this case, corneal allograft EC reparative response shows its own features related to its severe damage and cell derivation more so than other solid transplants.

The knowledge related to EC regeneration and replacement is valuable because it not only influences our understanding of graft characteristics but also helps in determining effective treatment approaches for protecting the graft. After transplantation, frequent therapy is the main means used to protect the graft and/or modulate immune response, a practice which could alter graft EC origin [14-16]. However, there has until now been no treatment in clinics for the lost transparency induced by rejection. We had transferred graft-protective, adenovirus-mediated nerve growth factor into the allograft ECs before transplantation in the rat model, which diminished the expression of pro-inflammatory cytokines, increased the expression of anti-apoptotic molecules, prolonged the graft survival significantly, and even resulted in zero rejection in some transplants that retained transparency. Furthermore, no cell in growth from the adjacent area was found at day 12 after the transplant and only a small proportion of ECs was lost [8]. The results encourage further study on corneal allograft EC repair and protection.

Our findings could contribute to establishing a proper direction for corneal therapy such as enhancing the reparative response and generating a functioning EC monolayer through the initiation of proliferation and replacement of host-derived ECs, protection of graft ECs, or building EC chimerism of graft and recipient. Since corneas can be stored ex vivo for a relatively long time before transplant, preconditioning treatment to protect the ECs to construct an accommodation status by using therapy such as local therapeutic gene transfection is one option [8,17]. Furthermore, the corneal graft is easily accessed following the operation so that topical treatment initiating EC chimerism or replacement could be applied locally not only on the graft but also on the recipient.

In conclusion, corneal allograft EC replacement represents a reparative response to transplant-related injury rather than a constant mechanism of tissue maintenance that depends on the regenerative features of ECs. These new findings should be beneficial for developing new strategies for corneal therapy.
